# Draft Genome Sequence of Microbacterium sp. Gd 4-13, Isolated from Gydanskiy Peninsula Permafrost Sediments of Marine Origin

**DOI:** 10.1128/MRA.00889-19

**Published:** 2019-10-03

**Authors:** Elena V. Spirina, Konstantin V. Kuleshov, Alfiya K. Yunusova, Tatiana A. Vishnivetskaya, Elizaveta M. Rivkina

**Affiliations:** a Institute of Physicochemical and Biological Problems in Soil Science, Russian Academy of Sciences, Pushchino, Russia; b Federal State Budget Scientific Institution Federal Scientific Center VIEV, Moscow, Russia; c Institute of Theoretical and Experimental Biophysics, Russian Academy of Sciences, Pushchino, Russia; d University of Tennessee, Knoxville, Tennessee, USA; Indiana University, Bloomington

## Abstract

Here, we report the draft genome sequence of Microbacterium sp. strain Gd 4-13, isolated from late Pleistocene permafrost of marine origin located on the Gydanskiy Peninsula. Genome sequence analysis was performed to understand strain survivability mechanisms under permafrost conditions and to expand biotechnology applications.

## ANNOUNCEMENT

Permafrost is the natural repository of a vast pool of ancient viable microorganisms that possess new enzymes for biotechnological applications (1, 2). Isolates obtained from marine permafrost on the Gydanskiy Peninsula (72°20′55.99″N, 78°32′48.50″E) were screened for the presence of endonuclease activity (3). Strain Gd 4-13 (VKM Ac-2796), which was derived from a depth of 4.0 m (4), belongs to the genus Microbacterium and shows the presence of type II restriction endonuclease (enzyme number 218866, http://rebase.neb.com/rebase/enz/MspGI.html). Despite the wide distribution of *Microbacterium* spp. in nature, the only cultivated close relative to strain Gd 4-13 is Microbacterium hatanonis FCC-01^T^ (GenBank accession number AB274908), obtained from hair spray (5). The cells of strain Gd 4-13 are nonmotile, non-spore-forming short rods distinguished from *M. hatanonis* based on their ability for growth at 10% NaCl and the synthesis of restriction endonuclease. Since the genome of *M. hatanonis* is not currently available, and there are no data on phenotypic diversity, metabolic activity, or adaptive survival strategies for closely related strains, we investigated the novel strain Gd 4-13 and its genome sequence.

An overnight culture of Gd 4-13 obtained in LB at 28°C with aeration was used for genomic DNA extraction with the UltraClean microbial kit (Mo Bio). A DNA library was prepared with the Nextera DNA library kit (Illumina) and sequenced on a HiSeq 2000 platform (Illumina). Adapter sequences and low-quality reads were trimmed using BBTools v.38.08 (https://sourceforge.net/projects/bbmap). *De novo* assembly was performed using SPAdes v.3.10.01 (6). Contigs below 500 bp with low k-mer coverage and taxonomic assignment outside *Actinobacteria* were considered contamination and were removed (7–9). Filtered and validated contigs were annotated using the NCBI Prokaryotic Genome Annotation Pipeline (10).

The draft genome assembly contains 20 contigs with an average coverage of 200×, a total length of 3,429,146 bp, an *N*_50_ value of 382,878 bp, and a high GC content of 69.4%. Genome annotation revealed a total of 3,268 genes, including 3,216 protein-coding sequences (CDSs), 54 pseudogenes, 1 rRNA (*rrn*) operon, 46 tRNAs, 3 noncoding RNAs (ncRNAs), and 25 genes encoding endonucleases. Analysis of the annotated proteins against the InterPro protein signature database using InterProScan v.5.23-62.0 revealed that the genome of *Microbacterium* sp. Gd 4-13 contains proteins involved in resistance to antibiotics and toxic compounds, many stress-related proteins such as oxidative stress proteins, and both cold shock and heat shock proteins.

Phylogenetic analysis of the complete 16S rRNA gene derived from the whole-genome sequence of strain Gd 4-13 revealed 100% identity to the uncultured bacterium SH201206-58 (GenBank accession number KX508409) and 99.6% identity to the type strain, *M. hatanonis* FCC-01 (GenBank accession number AB274908). The average nucleotide identities (ANIs) between the *de novo* assembled genome of *Microbacterium* sp. Gd 4-13 and 208 publicly available genomes from diverse strains of the genus *Microbacterium* were determined using BLAST+ and Pyani (https://github.com/widdowquinn/pyani) and varied from 72% (*Microbacterium* sp. KROCY2) to 78% (Microbacterium xylanilyticum JCM 13591), lower than the recommended cutoff point of 94% for species delineation (11–13). This fact supports the claim that strain Gd 4-13 may belong to a novel species of the genus *Microbacterium*.

### Data availability.

The raw sequencing reads have been deposited in the SRA (accession number SRX4514259), DDBJ Sequence Read Archive (DRA; accession number SRX4514259), and European Nucleotide Archive (ENA; accession number SRX4514259). This whole-genome shotgun sequencing project has been deposited at GenBank under the accession number QEIJ00000000. The version described in this paper is version QEIJ01000000.
